# Prevalence and Source of Fecal and Oral Bacteria on Infant, Child, and Adult Hands

**DOI:** 10.1128/mSystems.00192-17

**Published:** 2018-01-16

**Authors:** Michael Shaffer, Catherine Lozupone

**Affiliations:** a Department of Medicine, University of Colorado Denver, Aurora, Colorado, USA; b Computational Bioscience Program, University of Colorado Denver, Aurora, Colorado, USA; Luxembourg Centre for Systems Biomedicine

**Keywords:** computational biology, human microbiome, microbial ecology

## Abstract

Modern hygienic practices are applied to avoid exposure to pathogens that spread via fecal-oral transmission. Despite this, the gastrointestinal tract is quickly colonized by fecal microbes. The hands are an important vector for the transmission of microbes, but the frequency at which fecal and oral microbes exist on hands and the source of those microbes have not been extensively described. Using data from a previous study that characterized the fecal, oral, and skin microbiota from 73 families, we found a significant incidence of fecal and oral microbes on hands. Of palms, 48.9% had fecal signal and 67.2% had oral signal. Fecal, oral, and forehead microbes were tracked to family members and an individual’s own palms far more often than to unrelated individuals and showed relationships with age, gender, and parental status. For instance, oral microbes that were specifically sourced to the same individual (oneself) were most common on infant palms; mothers had more infant-child-sourced and oral-sourced microbes on their palms than nonparents. Fecal microbes on palms more often sourced to members of the family than unrelated individuals, but more often to other members of the family than oneself. This study supports that the hands are an important vector for the transfer of fecal and oral microbes within families.

**IMPORTANCE** Bacteria live all around us, and we are constantly exposed to them during our everyday lives. Modern standards of hygiene aim to limit exposure to fecal bacteria, and yet bacteria rapidly colonize the gut in early life and following antibacterial treatment. Exposures to fecal and oral microbes provide risk of disease, but are also necessary since commensal microbes play important roles in health. This work establishes that bacteria of both fecal and oral origins are commonly found on hands. It also establishes that the uniqueness of fecal and oral bacterial communities across people can allow for determination of the likely individual from whom the fecal and oral bacteria came. These techniques allow for understanding the hands as a vector for microbial transmission within families and across populations, which has important implications for public health.

## Abstract (precis)

Bacteria live all around us, and we are constantly exposed to them during our everyday lives. Modern standards of hygiene aim to limit exposure to fecal bacteria, and yet bacteria rapidly colonize the gut in early life and following antibacterial treatment. Exposures to fecal and oral microbes provide risk of disease, but are also necessary since commensal microbes play important roles in health. This work establishes that bacteria of both fecal and oral origins are commonly found on hands. It also establishes that the uniqueness of fecal and oral bacterial communities across people can allow for determination of the likely individual from whom the fecal and oral bacteria came. These techniques allow for understanding the hands as a vector for microbial transmission within families and across populations, which has important implications for public health.

## INTRODUCTION

After birth, humans rapidly acquire diverse communities of microbes all over the body, including on the skin and throughout the upper respiratory, urogenital, and gastrointestinal tracts, through exposure to the environment. The skin is the organ most exposed to the outside environment, and human skin-to-skin contact is a well-known pathway for bacterial transmission between individuals (1–3).

While the sharing of oral microbes via kissing has been documented (4), the vector by which the gut microbiome is transferred is less intuitive. Modern hygienic practices, including water treatment processes, sanitation of home environments, and hand washing, are widely applied in order to avoid exposure to pathogens that spread via fecal-oral transmission. Indeed, hand washing has been shown to have a strong effect on disease transmission (5, 6) and hand microbiome composition (7). Despite these practices, it is clear that the gastrointestinal tract is quickly colonized by fecal microbes in early life (8–10) and following antibiotic use (11, 12). These exposures are important, since a diverse microbial community in the gut promotes health (13–16) and since microbial exposures, particularly in very early life, are thought to protect against allergy and autoimmune disease (13, 17). However, gastrointestinal pathogens and pathobionts, including the stomach flu and *Helicobacter pylori*, readily spread across individuals, particularly among family members (18–21). Cohabiting nongenetically related individuals, such as domestic partners, share more similar fecal microbiomes than noncohabiting individuals (22). The degree to which this is driven by a direct sharing of microbes versus other factors, such as a shared diet, is not well understood.

The hands are known to be an important vector for the transmission of microbes, including pathogens, as hands commonly touch shared surfaces as well as our own mouths and each other’s bodies (23). Thus, perhaps more than any other site on the human body, the microbiota observed on any individual’s hands may be a combination of commensal bacteria that colonize the skin and bacteria from other sources that are determined by behaviors (such as gardening, nail-biting, or cooking) and interpersonal interactions (such as child care or intimate contact). Although multiple studies have characterized the microbiome of hands (24–26), the degree to which hand microbiomes may harbor fecal or oral bacteria and their origin are not well understood.

Individuals leave a microbial aura around them in their homes, and their contribution to the home microbiome diminishes with their absence (27). However, the extent to which palms act as a vector between individuals who are in common contact such as family members is not well understood. One previous study characterized the fecal, oral, and skin (hand and forehead) microbiota from 73 families (22) and found that the skin (palm and forehead) microbiome was more similar between individuals in cohabiting families than between individuals in different families and that dog ownership significantly increased the shared skin microbiota in cohabiting adults. Very little attention in this study was given to the degree to which microbes on the palms of these individuals may have come from fecal and oral sources and to whether fecal and oral bacteria on palms may be from oneself versus other individuals.

Fecal and oral microbiota display a strong degree of interpersonal variation, which allows the estimation of whether a palm microbiome contains fecal or oral bacteria generally and, to some extent, whether those bacteria were specifically from a particular individual. For instance, fecal microbes on an individual’s palm could be from their own feces or the feces of a member of their family. The SourceTracker software uses a Bayesian approach to estimate the proportion of microbes in a given sample that come from any number of possible source environments using 16S rRNA sequence data and has been used previously to estimate levels of fecal and oral contamination on surfaces (28).

In this study, we use data from this previous study that characterized the fecal, oral, and skin (hand and forehead) microbiota from 73 families. We first establish that SourceTracker can effectively be used to source sequences not only to stool or oral samples generally, but specifically to a sample from a particular individual if the oral/fecal microbiome composition of that individual is known. We next applied SourceTracker analysis to these data to understand: (i) the frequency at which microbes of fecal and oral origin are observed on hands, (ii) the degree to which fecal and oral microbes on hands have come from the individual, a person within an individual’s family, or an unrelated individual, and (iii) whether the prevalence and origin of fecal or oral microbes on hands differ across age groups or whether the microbes are most prevalent in particular intrafamily relationships (e.g., parents to children or between partners). A surprisingly high incidence of fecal material was found on hands that could be specifically tracked to that of family members and oneself, supporting that the hands are an important vector for transfer of fecal microbes within families.

## RESULTS

### Validation of SourceTracker mapping to sites and individuals.

**FIG 1:**
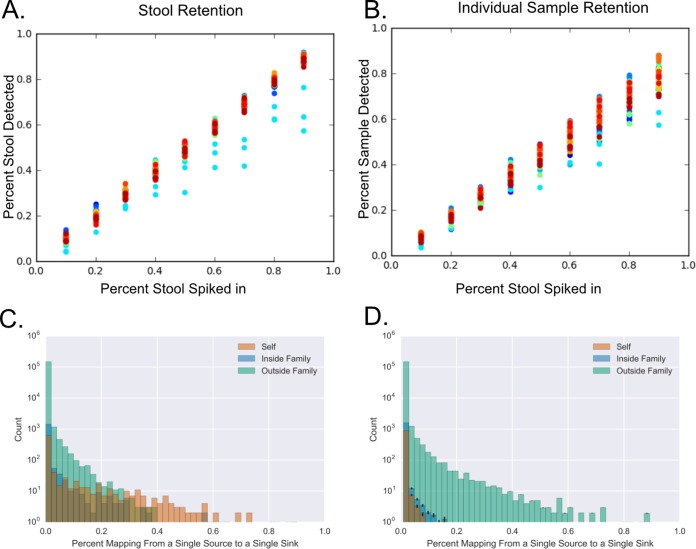
Validation of SourceTracker detection of microbial sequence origins. (A and B) Spike-in experiment in which reads from a single stool sample were added to the reads of palms that otherwise had no fecal signal. The degree to which the added (“spiked-in”) reads were assigned to any fecal sample (A) or the specific stool sample that was spiked in (B) using our SourceTracker protocol is shown. The plots show the results for 20 palms, each with 3 repetitions. Histograms show the count of sample source pairs (e.g., a single stool, oral, or forehead sample) to individual sinks (e.g., a single palm) with percentage mapping separated into self, inside-family, and outside-family sources as measured (C) and with randomly permuted data (D). In each permutation, reads from each palm were randomly assigned across all source samples, and this was repeated 25 times. Error bars represent the standard error measured across these permutations.

We determined the ability to specifically assign microbes from fecal and oral samples to a particular individual. A spike-in analysis was performed in which 20 palm samples that had little to no fecal or oral signal were “spiked” with reads from the stool or tongue sample from the same individual (i.e., to simulate that individual’s own stool or tongue bacteria being present on their hands). For each of 20 palm samples, the analyses were performed at 10, 20, 30, 40, 50, 60, 70, 80, and 90% spiked-in reads, and 3 replicates were done at each level. SourceTracker was then run with the samples from all individuals in the study as individual sources. On average, across replicates and samples, 97.5% of spiked-in stool sequences were tracked to any stool sample (Fig. 1A) and 86.5% were tracked to the spiked-in stool sample of that specific individual (Fig. 1B). With tongues, 97% of spiked-in sequences were tracked to any tongue sample, on average, and 81.9% tracked specifically to the spiked-in tongue sample (data not shown).

These spike-in analyses may overestimate the degree to which assignment to a particular individual can be made in the face of variability—e.g., in the bacteria present in different fecal samples of the same individual. To assess whether the assignment of sequences to samples of particular individuals is meaningful, we determined whether bacteria on a person’s palm are more likely from their own fecal, oral, or forehead samples or those of someone in their family than from an unrelated individual. The distribution of sequences on palms, predicted to originate from fecal, oral, or forehead samples from the individuals themselves or their family, was highly significantly different from a null distribution where every read was given an equal chance to be in any sample regardless of family membership status (Fig. 1C; *P* < 10^−160^). To visualize this difference, we randomly permuted the read sources on each palm 20 times and formed the same plot as with the real data (Fig. 1D). This clearly shifts the distribution, as expected, since samples from outside the family of a particular individual vastly outnumber the samples from inside this individual’s family and the samples from their own person. Analysis of family origin was repeated individually for the forehead, stool, and tongue sites, and assignment of sequences to oneself or family was found to be highly significantly different from the null distribution (all *P* < 10^−160^) for all 3 body sites.

### A single family shows cross exposure of bacteria.

**FIG 2:**
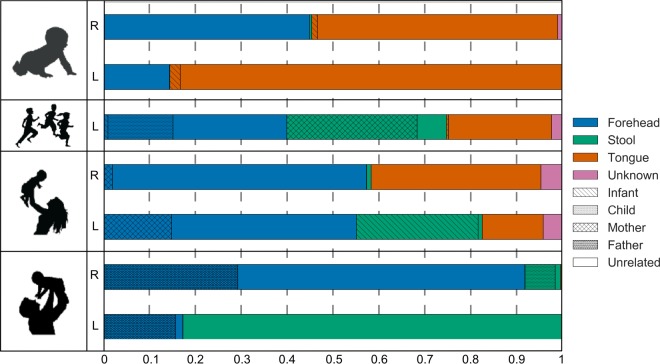
The result of running the SourceTracker analysis on the palms of a single family. The predicted proportions of bacteria on the right (r) and left (l) palms of a family consisting of an infant (1 year old), child (3 years old), mother, and father that are from the forehead (estimated by forehead samples from the same individuals), stool, tongue, and unknown source are shown. Additionally, the hatching pattern indicates the predicted proportion within each of these sources that is specifically from the infant, child, mother, or father.

To show the utility of our approach, a single family consisting of a mother, father, child, and infant was first analyzed (Fig. 2). In addition to using oral and fecal samples as sources, forehead samples were used to estimate the degree to which bacteria observed on the hand may be commensal skin bacteria. As expected, forehead bacteria were the most prevalent “source” of bacteria on hands, composing an estimated 14.5 to 91.8% of the microbes across palms. Of these forehead bacteria, 1.5 to 43.9% specifically mapped to a particular individual in the family rather than to any of the other 172 individuals in the study, which is significantly higher than expected by chance (*P* < 10^−5^). In the case of forehead microbes that tracked inside the family, the source was almost exclusively oneself rather than a family member.

The infant’s hands were dominated by tongue bacteria (53.6 and 85.5%). The mother and child of the family also had a high proportion of tongue bacteria on their palms (13.3 to 37%). Tongue bacteria did not often track to any particular individual in the family or oneself; 1.2% and 2.3% of tongue bacteria on the infant palms mapped to the infant, which is low but still significantly higher than any other individual in the study (*P* < 10^−25^).

Strikingly, the father, mother, and child all had fecal microbes (ranging from 1.1 to 82.6%; median, 27.4%) on at least one of their palms. The source of stool bacteria on the palm was more often from a family member than expected by chance, but from other family members and not necessarily oneself (*P* < 0.01). For instance, 81.5% of the fecal signal on the child’s left palm was predicted to be from her mother and 96.9% of the fecal signal on the mother’s left palm was predicted to be from her infant. These observations are consistent with what would be expected if the sequences were entirely from that single fecal source sample, as measured in our spike in experiments, and are higher than those observed in unrelated individuals (all *P* < 0.001). On the father’s left palm, none of the 82.6% of bacteria that tracked to stool generally was his own or from a family member. Taken together, the analysis of this particular family indicates a high degree of oral and fecal signal on palms that can often be tracked specifically to fecal or oral samples of oneself or family members, which further substantiates a true human fecal or oral origin.

### Broader analysis of the full cohort.

**FIG 3:**
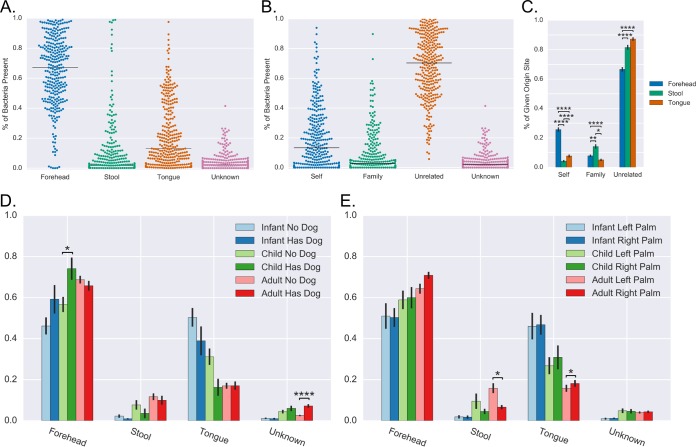
Distributions of samples across sources. (A) Percentage of bacteria present on each palm that sources to forehead, stool, tongue, or unknown sources. The forehead, stool, and tongue values are the sum of those mapping to all individual forehead, stool, or tongue samples in the whole cohort. Each point in each column is an individual palm, and each palm is represented in each column. (B) Percentage of bacteria present on each palm that source to forehead, oral, and fecal samples from oneself (the same individual as the sink), members of their family, or unrelated individuals in the study. Each point in each column represents a single palm, and each palm is represented in each column. (C) Average proportions of sequences mapping to oneself, family members, and unrelated individuals in reads that map to forehead, stool, and tongue sources. (D) Average proportions of sequences that map to a given body site based on dog ownership. (E) Average proportions of sequences that map to a given body site from a left or right hand of individuals from a given age group. Error bars show standard error. *, *P* < 0.05; **, *P* < 0.01; ****, *P* < 0.0001.

Having established fecal and oral signal on palms that could be tracked to specific family members in one family, the set of 73 families (172 individuals) were evaluated. Most palms have a high abundance of bacteria from forehead sites (Fig. 3A). Across all individual palms, the median amount of stool was 1.7%, with a range of 0% to 98.9%. Overall 48.9% of palms (from 62.7% of individuals) had fecal signal above noise (as estimated based on SourceTracker-provided estimate standard deviations) and 36 (11.8%) hands had over 25% fecal signal. Oral bacteria was even more prevalent than stool, with oral bacteria making up a median of 13.1% of reads on palms (Fig. 3A); 67.2% of palms (from 81.9% of individuals) had oral signal above noise.

The degree to which bacteria on palms could be traced specifically to other members of the study was determined (Fig. 3B). The sources were divided into three groups for *post hoc* analysis for each palm: oneself, family, and unrelated (nonfamily) (Fig. 3B and C). In Fig. 3B, each point represents a palm, and the “Self” column sums the percentage of reads that mapped to the forehead, feces, or tongue of that individual, the “Family” column sums those that mapped to all samples from that person’s family, and the “Unrelated” column sums those that mapped to samples from all other unrelated individuals in the study. Although the majority of bacteria on palms mapped to unrelated individuals in the study (median value of 70.4%), self-sourced bacteria made up a median value of 13.3% of the bacteria on palms and family-sourced bacteria 2.0%. The proportions of bacteria that mapped to oneself, family, or unrelated for each source site were also summarized (fecal, oral, and forehead) (Fig. 3C). Tongue and forehead bacteria detected on palms were more often from oneself than from a family member, but fecal bacteria were more often from a member of an individual’s family than themselves.

### The origin site and specificity of origin of hand bacteria change with age.

**FIG 4:**
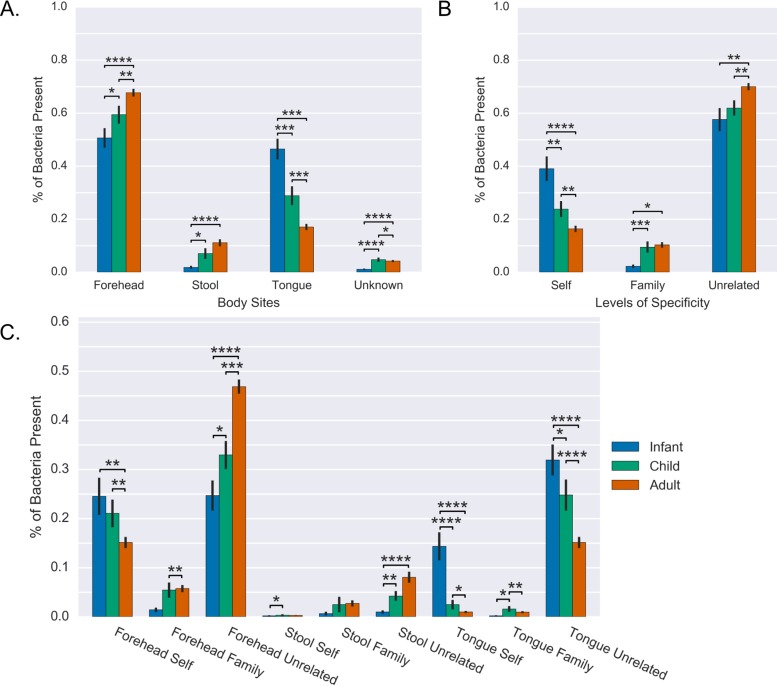
Shifts in bacterial origin with age. (A) Average percentage of bacteria present on palms from different body sites broken up by age. (B) Average percentage of bacteria present on palms from oneself, family, and unrelated sites broken up by age. (C) Average percentage of bacteria present on palms from all combinations of body site origins and self, family, or unrelated origins broken up by age. Error bars show standard error. *, *P* < 0.05; **, *P* < 0.01; ***, *P* < 0.001; ****, *P* < 0.0001.

This data set includes families with individuals between the ages of 6 months and 87 years, and we sought to find if age had an effect on palm source composition. The data set was divided into the three groups infants (age, <2 years), children (age, >2 and <18 years), and adults (age, >18 years), and the proportions from forehead, fecal, and oral sources across age groups were examined. The predicted proportion of bacteria from the tongue was higher on the palms of infants compared to children (*P* = 0.02) and children compared to adults (*P* = 0.008) (Fig. 4A). Corresponding with these decreases, the amount of bacteria sourced to stool (infant to child, *P* < 0.05; child to adult, not significant [NS]) and forehead (infant to child, *P* < 0.05; child to adult, *P* < 0.01) increased with age.

Differences associated with age class in the proportion of bacteria on palms predicted to be from an individual’s own body sites, body sites from other individuals in the family, or body sites from the general population were tested. The predicted proportion of bacteria from samples from oneself decreased significantly with age, with infant palms being 39.0% self specific on average and rates of 23.8% for children and 16.3% for adults (Fig. 4B). Corresponding with these decreases, the amount of bacteria tracking to family members or unrelated individuals significantly increased with age (Fig. 4B). However, by further breaking down sources into site and specificity groups (Fig. 4C), it is clear that the increase in self-mapping bacteria in infants is largely driven by the presence of more of their own tongue bacteria on their palms. Since these are relative and not absolute abundances, the decrease in the proportion of fecal or forehead bacteria on infant hands and in nonself sources may be driven by an increased load of bacteria from their own saliva.

We sought to associate the body site origin of bacteria on palms with available metadata from the original study. Differences in body site origin of bacteria between left and right palms were determined in different age categories (Fig. 3E). Adult left palms had significantly more stool bacteria (*P* = 0.01) than right palms and less tongue bacteria (*P* = 0.04). No significant differences between left and right palms were found in infants and children. Handedness information was not gathered in this study, so we were unable to assess if this result is driven by handedness of individuals in the cohort. We also investigated the effect of dog ownership on body site origin of palm bacteria (Fig. 3D). There was a significant increase in microbes of unknown origin (*P* < 0.001) in adults with dogs, which is expected since dog samples were not included as sources here, but were identified in the original study as a source of microbes on skin (22). A significant increase in forehead-mapped bacterial reads was also found in children with dogs, which may indicate some assignment of dog-sourced microbes to forehead.

### Gender and parental status influence source of bacteria on hands.

**FIG 5:**
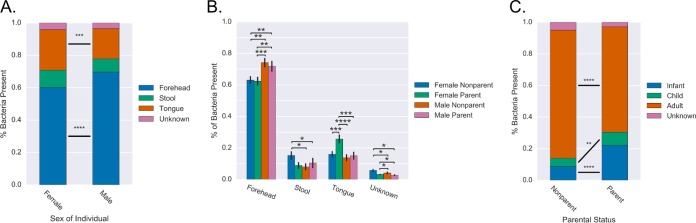
Gender and parent status affects palm bacterial composition. (A) Average profile of women’s and men’s palms in terms of bacterial site origin. (B) Average proportion of bacteria from origin sites in males and females further broken down by parental status. Only adults are included in this analysis. Error bars represent the standard error of the mean. (C) Average profile of parent and nonparent palms in terms of age group and origin of sample for tracked bacteria. *, *P* < 0.05; **, *P* < 0.01; ***, *P* < 0.001; ****, *P* < 0.0001.

Gender also had an effect on the body site source composition of palm bacteria. Male palms had significantly more bacteria map to forehead sources than female palms (69.5% versus 59.6%; *P* < 10e^−5^) (Fig. 5A). Females had significantly more (*P* = 0.004) bacteria mapping to the tongue present on their palms (25.3%) than males (19%) (Fig. 5A). To check for age bias, adult samples were analyzed independently. Adult male palms maintained a significantly higher proportion of bacteria mapping to forehead than women (*P* = 0.0002) and significantly less bacteria from all other sources (stool, *P* = 0.01; tongue, *P* = 0.003; unknown, *P* = 0.004).

The effects of parent status were also determined. Women who were not parents had more stool and unknown bacteria (both *P* < 0.05) and less forehead bacteria (*P* < 0.001) than men who were not parents. There was a significant increase in the proportion of tongue bacteria on palms of adults who were parents compared to nonparents (*P* = 0.001), and this was driven primarily by a significant increase in mothers (*P* < 0.001), while the change in fathers was insignificant (Fig. 5B). Men had no change in bacterial origin with parent status. Women who were not parents did not have more oral microbes on their hands than nonparent men, supporting that exposure to children may be the driver of an increase in oral signal on the parent’s, specifically mother’s, palms. Consistent with this hypothesis, nonparent adult palms had a median 6.4% of bacteria coming from child-infant sources, while parent’s palms had 26.5% of bacteria from child-infant sources, a significant difference in values (*P* < 10^−9^) (Fig. 5C).

## DISCUSSION

Our results suggest that despite modern hygienic practices, the palm very frequently carries both fecal and oral microbes. Many results are intuitive. It is not surprising to find that infants have a high incidence of their own tongue bacteria on their palms, as they frequently put their hands in their mouth. Similarly, the detection of self-specific tongue bacteria on adult hands is consistent with adult individuals touching their own mouths, but with less frequency than infants and children. Parents had higher proportions of bacteria from infant and children on their palms, and mothers in particular also had a higher incidence of tongue bacteria on their palms, indicating that hand-to-hand contact with infants/children harboring high oral signal on their hands may be another source of oral microbes on adult hands.

That fecal signal was found on 48.9% of evaluated palms (from 62.7% of individuals), with 11.8% having over 25% fecal signal, is perhaps a surprisingly high incidence, since hygienic practices such as sanitation of home environments and hand washing are widely used. However, this result may be consistent with the observation of rapid colonization of the gut by fecal microbes in infants (8–10) and following antibiotic use (11, 12) and may indicate that the transmission of fecal microbes between individuals via the hands is more frequent than commonly thought. It follows that either fecal exposure is frequent or hand washing is not completely effective at removing fecal microbes from hands. Our results also suggest that the previous observation that cohabiting nongenetically related individuals, such as domestic partners, share more similar fecal microbiomes than noncohabiting individuals (22) may be in part driven by a direct sharing of microbes as well as other factors such as a shared diet.

Handedness might be expected to influence the presence of oral and fecal microbes on hands; for instance, the dominant hand may be more exposed to fecal microbes during hygiene after defecation or oral microbes during cosmetic applications or the covering of a cough or sneeze. Although this data set did not include information on the handedness of individuals, an estimated 90% of the U.S. population is right-handed, and there indeed were significantly greater levels of oral microbes on the right hands of adults. This trend was not true for infants, which may be related to infants putting dominant and nondominant hands in their mouth indiscriminately. Surprisingly, there was a significantly increased proportion of fecal microbes on left compared to right palms, a result we find hard to interpret.

Adults have a higher proportion of bacteria from stool and a lower proportion of self-mapping bacteria on their palms compared to children and infants. One potential explanation is that adults may interact more with individuals outside their immediate family and/or surfaces outside the home that may harbor fecal microbes. However, since these are relative and not absolute abundances, these trends may be driven by a high load of self-mapping oral bacteria on infant palms, which would result in proportionately less bacteria from other sources. Quantitative information would be needed to distinguish between these two potential explanations.

Consistent with previous studies reporting gender effects on microbiomes, including skin microbiomes (7, 29–32), a significant effect of gender on the sources of bacteria on palms was found: for instance, adult women had more oral bacteria on their palms than men, and men had higher proportions of forehead bacteria. Palms with higher percentages of bacteria mapping to forehead (rather than fecal, oral, or unknown sources) may indicate signal dominated by commensal bacteria rather than bacteria from other sources. Parenthood had an effect on the origin of bacteria on the palm in females and not males, with mothers and not fathers having increased proportions of tongue-sourced bacteria. This is consistent with women having more child care responsibilities (33, 34). However, gender differences cannot be completely explained by child care; the palms of nonparent males had less stool and unknown bacteria and more forehead bacteria than the palms of nonparent females. These differences could possibly be explained by differences in behaviors such as cosmetic application that leads to increased face touching as well as differences in hygienic practices.

Our analysis has several potential weaknesses. First, the 16S rRNA sequence data do not give information on bacterial load on palms. Additionally, forehead samples were used to estimate the degree to which bacteria observed on the hand may be commensal skin bacteria. However, we cannot rule out the possibility that bacteria assigned to forehead (“skin”), or any other body site, may also include bacteria from other sources (exposures) that are shared between those sites (e.g., as may happen if an individual rested their palm on their forehead). Furthermore, previous studies have found significant differences between dry skin sites such as the palm and sebaceous skin sites such as the forehead (35), so bacteria that only colonize dry skin sites may not track to our forehead sites. It is also difficult to determine the degree to which false positives are present in our analyses; e.g., bacteria from related environments such as the vagina may map to stool because the actual source was not in our analysis. However, the highly significant degree to which fecal and oral mapping came from oneself and family members rather than unrelated individuals indicates a high degree of true human fecal/oral origins.

Our spike-in experiments suggested a high ability to map fecal and oral sequences specifically to a single individual. This result indicates that a high degree of interpersonal variation in samples allows for specific mapping of stool and oral samples to particular individuals. This highly specific assignment in our spike-in simulations may be hard to reconcile with high levels of mapping to unrelated individuals in our analyses across the whole cohort. Since we do not expect that there was direct contact with unrelated individuals, the high degree of assignment to the “unrelated” category is most likely driven by the presence of shared microbes across the population. Although our spike-ins show a strong ability to assign sequences to the correct sample when that sample is in the data set, nonspecific assignment will occur when the true source is not present because, even if the operational taxonomic unit (OTU) is not common, it is likely to be found in at least one other person in the population. For instance, looking at shared OTUs, we found that only 34 of 73 families had an OTU that was specific to that family and that ~21% of OTUs are present in at least 10% of all samples. In our spike-in simulations, specific mapping of a sequence to the spiked-in sample was not as high for oral as for fecal samples. A possible reason for this is the higher levels of conservation of the oral microbiome across individuals, as has been reported in other studies (25).

Overall, we also do suspect that the high degree of mapping in our spike-in simulations overestimates our ability to assign sources to individuals in the real data because there is variability in the microbiomes of individuals over time and within body sites (e.g., in different parts of the mouth or within a single stool sample). Thus, we believe that the reported proportions of bacteria that are from oneself and family oral and fecal samples versus those of unrelated individuals are underestimates.

Replication of this study via deep metagenome sequencing or even deeper 16S sequencing with longer read lengths to get more taxonomically specific abundance data could help better differentiate closely related taxa, which may show diversity at the individual and family level. Additionally, the sampling done in the original study included only a single time point. A longitudinal analysis would allow determination of which bacteria were present based on transient exposure and which were more long-term colonizers. Additional metadata could help in the analysis of these data. Understanding of hand washing frequency and handedness could be factors that affect bacterial composition on hands. Knowing child care responsibility and if unrelated individuals in the study shared workspace could help elucidate additional relationships between individuals. A study such as this in a hospital setting could find potential transmission of microbes from caregivers to patients and vice versa, as has been found in studies after childbirth (36–38).

The original study that described these data also used SourceTracker to estimate human fecal and oral signal on hands, but differed in that all samples of the same type (e.g., feces) were defined as the same “source,” rather than defining each sample as a unique source. Our approach uniquely allowed for evaluation of fecal/oral bacterial origins by family relationships. It is worth noting, however, that higher overall fecal and oral signal is reported here than was originally reported. Specifically, the original paper reported that on average, ~11% of the palm community originated from human oral sources, which is much lower than our 21.9% average. Similarly, their average of <2% stool bacteria per palm is much less than our 9.5% average.

These differences are likely driven by a preprocessing step that was performed in the original paper but not here, in which samples were removed if they did not classify to their own site type with a random forest classifier (e.g., a sample that was labeled as palm but predicted to be of oral origin was excluded). This filtering step, in addition to removal of samples with <5,000 sequences per sample (compared to 1,000 sequences per sample in our analysis), resulted in the exclusion of 107 samples (~10% of the total). This random forest filtering step was applied to remove samples that may have been mislabeled by study participants, which is a reasonable approach when subjects are collecting samples at home without supervision. However, the findings of our study suggest that mislabeling is not the likely cause of samples being classified inappropriately. For instance, we found that infants in particular had large proportions of oral bacteria present on their palms. This observation is more easily explained by infant behavior than by infant palm samples being preferentially mislabeled.

The overall results of these analyses seem to follow common wisdom. Palms are the most exposed parts of the body, exposure to other body sites is common, and this can be seen in their microbial community being of diverse origin. These results show that exposure seems to play a part in forming the bacterial communities present and that family membership and structure play a part in determining the composition of palm communities. This exposure and route of transmission could have effects on sharing of microbes in clinical and other settings with importance worthy of future study.

## MATERIALS AND METHODS

All analyses done in this study are available in the form of Jupyter Notebooks at https://github.com/lozuponelab/Source_of_Fecal_and_Oral_Bacteria_on_Hands.

### Data set.

The data sets used were downloaded from qiita.microbio.me. The Song et al. family study (Qiita study ID 797) was used for the analyses (22). Only human palm, tongue, and stool samples were used in the analysis. Samples were sequenced using bar-coded sequencing of the V2 hypervariable region of 16S rRNA on the Illumina GAIIx. Reads were clustered at 97% using SortMeRNA (39) and the greengenes database (40) by Qiita.

### SourceTracker analysis.

The tool SourceTracker was used for the majority of these analyses (28). The table was first filtered to remove samples with less than 1,000 reads. SourceTracker was run with all palms set as sinks and all other samples set as individual sources and default settings.

### Spike-in analyses.

Twenty palm samples were spiked with reads from the stool or tongue sample from the same individual. For each sample, spike-ins were performed at 10, 20, 30, 40, 50, 60, 70, 80, and 90% spiked-in reads and three replicates were done at each level. When performing spike-ins, the entire read population per sample was used and the number of reads to be spiked in was calculated with the equation *R* = [*S*/(1 − *r*)] − *S*, where *R* is the number of reads to be added to the sample, *S* is the total number of reads in the starting sample, and *r* is the desired final ratio of spiked reads to total reads. The *R* reads were then randomly selected from the source sample (that person’s own tongue or stool sample) and added to the original sample. This sample was then rarefied down to 1,000 reads and used as the input sample for SourceTracker.

### Statistical analyses.

To test for enrichment of assignment to oneself and family sites versus unrelated sites, the Cochran-Mantel-Haenszel test was performed using R (41, 42). Each palm was considered an independent trial, and observed counts for reads mapped to self samples, in-family samples, and out-of-family samples were counted as one group. The other (“null”) distribution was determined by randomizing the sequences such that each read had an equal chance to be assigned to any sample and then self, in-family, and out-of-family counts were determined per sample. The average null distribution reported in Fig. 1D represents 25 random permutations, and the standard error across permutations was calculated.

For the initial analysis, a single family (family 2) was selected from the Song et al. data set. To test if significantly more bacteria were being mapped to members of family 2 compared to outside mappings, a Mann-Whitney *U* test was used to compare the proportion of reads mapped to samples from unrelated individuals to the proportion of reads mapping to individuals inside the family.

To test for greater than 0 content from fecal or oral sources on palms, a *t* test was performed on the mean and standard deviation of the SourceTracker predictions with an alpha value of 0.05. In all other statistical tests, source samples were summed based on metadata grouping (e.g., body site origin or family sample origin). To compare source groupings between sink groupings (e.g., parent status or age groups) a Mann-Whitney *U* test, as implemented in SciPy (43), was used to compare groups.

Visualizations were designed with pandas (44), matplotlib (45), and seaborn.

### Data availability.

The raw data used in this study are available at the European Nucleotide Archive under study no. PRJEB1799. Additionally, all study data and the OTU table used in our analyses are available in the QIITA database at https://qiita.ucsd.edu/study/description/797.
